# Angiolymphatic invasion as a prognostic fator in resected N0 pancreatic adenocarcinoma

**DOI:** 10.1590/0102-6720201700010012

**Published:** 2017

**Authors:** Ricardo Vitor Silva de ALMEIDA, Adhemar Monteiro PACHECO-JR, Rodrigo Altenfelder SILVA, André de MORICZ, Tércio de CAMPOS

**Affiliations:** Discipline of Surgery of the Pancreas and Biliary System, Department of Surgery, Brotherhood of Santa Casa de São Paulo, São Paulo, SP, Brasil.

**Keywords:** Pancreatic neoplasms, Adenocarcinoma, Outcome assessment.

## Abstract

**Background:**

Pancreatic adenocarcinoma remains one of the worst digestive cancers. Surgical resection is the main target when treating a patient with curative intent.

**Aim:**

To assess angiolymphatic invasion as a prognostic factor in resected pN0 pancreatic cancer.

**Methods:**

Thirty-eight patients were submitted to pancreatoduodenectomy due to head pancreatic cancer. Tumor size, margins, lymph nodes, pTNM staging, angiolymphatic and perineural invasion were described in the pathologists' reports.

**Results:**

Most patients were female. Overall median survival was 13 months. Gemcitabine was the regimen of choice for chemotherapy in selected patients; however, it did not improve overall survival. pR0 resection had better survival compared with pR1. Within the pN0 group, survival was significantly better in patients without angiolymphatic invasion.

**Conclusion:**

Angiolymphatic invasion in N0 pancreatoduodenectomy can be demonstrated by the Hematoxylin-Eosin stain and may predict a poor prognosis factor for those patients.

## RESUMO

**Racional:**

Adenocarcinoma pancreático continua sendo um dos piores cânceres do aparelho digestivo. A ressecção cirúrgica é o principal objetivo quando se trata de intenção curativa.

**Objetivo:**

Avaliar a invasão angiolinfática como um fator prognóstico no câncer da cabeça do pâncreas ressecado pN0.

**Método:**

Trinta e oito pacientes foram submetidos a duodenopancreatectomia por câncer da cabeça do pâncreas. Tamanho do tumor, margens, linfonodos, estadiamento pTNM, invasão angiolinfática e perineural foram descritos nos laudos anatomopatológicos.

**Resultados:**

A maioria foi de mulheres. A sobrevida mediana global foi de 13 meses. Gencitabina foi a droga de escolha para quimioterapia nos pacientes selecionados, entretanto não aumentou a sobrevida global. Pacientes com ressecção pR0 tiveram sobrevida global superior quando comparados com ressecção pR1. Dentro do grupo de pacientes com pN0, a sobrevida foi significativamente melhor no grupo de pacientes que não apresentavam invasão angiolinfática.

**Conclusão:**

A invasão angiolinfática da duodenopancreatectomia N0 pode ser demonstrada utilizando apenas a hematoxilina-eosina e pode predizer prognóstico ruim para estes pacientes.

## INTRODUCTION

Surgical resection remains as the only possibility for the complete cure of patients with cephalic pancreatic adenocarcinoma. Such disease is the fourth leading cause of cancer-related mortality, with a median survival of 5-8 months, 5-year overall survival of less than 5% considering all stages of the disease, and 20% of those treated with curative intent^11^. In Brazil, it is responsible for 2% of all types of cancer and 4% of all cancer-related deaths. Therefore, this disease has the poorest overall survival amongst all other types of cancer.

It is a rare condition before the age of 45 years, mostly occurring after de sixth decade. Therefore, with population aging in western world, its incidence tends to rise in absolute numbers. Even after potential surgical curative resection, about 80% of the patient dye of disease due to distant metastasis or local recurrence^29^. The rate of recurrence is predetermined by the microscopic frequently incomplete resections as a result of anatomical tumor location and growth pattern of cancerous cells^6^ ^,^ ^28^. Several factors contribute to a better or poorer oncologic prognosis after resection surgery in these patients. Among them there are tumor size, degree of cell differentiation, lymph node status, margins/R status, and CA19.9 levels^21^ ^,^ ^32^.

Staging for pancreatic ductal adenocarcinoma has been proposed by the Japanese Pancreas Society and the Union for International Cancer Control (which is the same as the American Joint Committee on Cancer - AJCC). These staging systems have a similar TNM classification. However, they considerably differ in the final clinical stage grouping^10^. These TNM staging systems have proven to be poor in predicting long-term overall survival when analyzing resected pancreatic adenocarcinoma, providing only an anatomical analysis for the extent of the disease^2^.

Therefore, there is a necessity to identify determinant factors/variables in long-term overall survival, and these factors are based on histopatological examination.

The invasion of tumor cells into lymphatic or blood vessels (angiolymphatic invasion) is crucial for the metastatic process. This feature is routinely studied and demonstrated in pathologists' reports using the HE stain only. It has been shown to have clinical impact in overall survival not only in periampullary but also colorectal, gallbladder, pancreatic pseudopapilary tumor, and breast cancers^4^ ^,^ ^7^ ^,^ ^14^ ^,^ ^15^ ^,^ ^20^. Several papers have been found regarding lymphovascular invasion as a prognostic factor in periampullary cancer. Nevertheless, most of them included not only pancreatic adenocarcinoma, but also other types of tumors, such as common bile duct, ampulla of Vater carcinomas, pancreatic pseudopapillary, gallbladder and even pancreatic neuroendocrine tumors. None of them have studied only N0 pancreatic adenocarcinomas^4^ ^,^ ^5^ ^,^ ^12^ ^,^ ^15^ ^,^ ^16^.

The aim of this study was to assess the angiolymphatic invasion as a potential prognosis factor in resected N0 pancreatic adenocarcinoma.

## METHODS

This research had been approved by the institution's human research ethics committee (#05625612.4.0000.5479). It is a retrospective cohort held in patients who underwent pancreatoduodenectomy of head pancreatic cancer at the Central Hospital of Santa Casa de S*ã*o Paulo, School of Medical Sciences, São Paulo, SP, Brazil, a tertiary academic institution, from 2000 to 2013. Data were prospectively retrieved from patients' records both from outpatient clinics and inpatient care, image and laboratory examination results, and operation reports, using the current definitions in pancreatic surgery.

Inclusion criteria: patients submitted to classic or pylorus-preserving pancreatoduodenectomy due to pancreatic adenocarcinoma; and Karnofsky Performance Status >or=80%. Exclusion criteria: pancreatoduodenectomy performed due to benign diseases such as chronic pancreatitis, serous or mucinous cystadenoma, neuroendocrine tumor, pancreas divisum and pancreatic solid pseudopapillary neoplasm; carcinomas from the ampulla of Vater, distal choledochus, and duodenum; locally advanced or metastatic disease; KPS<80%; patients that were not adequately followed in outpatient clinics after surgery.

Population characteristics were analyzed (age, gender), time until diagnosis, serum bilirubin levels, surgical approach and time length, types of resection and reconstruction, postoperative complications, postoperative pancreatic fistula, delayed gastric emptying, pathologic staging, histopathological aspects, adjuvant therapy, overall survival and causes of death.

Pathologists experienced in pancreatic diseases in the institution analyzed the specimens. Reports included both macroscopic and microscopic description, were based on HE stain, and included tumor size and tumor invasion, degree of tumor-cell differentiation, assessment of surgical margins (especially retroperitoneal and distal pancreatic margins), pR status (considering pR1<1 mm), lymph node, angiolymphatic and perineural invasion. pTNM status according to the UICC was determined. Immunohistochemistry was used to determine the exact origin of the tumor when there was doubt. 

Adjuvant chemotherapy or chemoradiation was based on gemcitabine regimen and was indicated for those patients with N+ status, non-R0 status, and T3 tumors.

In order to standardize definitions for postoperative pancreatic fistula (POPF) and delayed gastric emptying (DGE) from the International Study Group in Pancreatic Surgery (ISGPS), these data were prospectively analyzed using the online calculator available at http://pancreasclub.com/calculators/isgps-calculator/ ^1^.

### Statistical analysis

Was done using IBM SPSS Statistics v.21. Chi-square test was used for categorical variable comparisons, Pearson correlation, and Log-Rank/Mantel Cox test for nonparametric variables. p< 0,05 and CI 95% was considered as a significance.

## RESULTS

A total number of 310 patients were diagnosed with cephalic pancreatic cancer in the Division of Pancreatic and Biliary Surgery outpatient clinics within the period of 2000-2013. The great majority was not elected to surgery with curative intent due to locally advanced or metastatic disease by the time of the diagnosis, so that only 38 patients could be submitted to pylorus preserving pancreatoduodenectomy (PPPD) or classic pancreatoduodenectomy (CPD) and adequately followed after surgery. 

Sixteen patients were male and 22 female. Median age was 60 years (32-83). Main symptom was jaundice with a median time of 30 days (0-180) prior to diagnosis. Median total bilirubin level was 15.6mg/dl (0.2-38.0). Only two patients had endoscopic biliary drainage prior to operation because of inconclusive diagnosis (Table 1).

**TABLE 1:** Epidemiologic data from patients submitted to pancreatoduodenectomy due to head pancreatic cancer

Patients	Enrolled	38
	Excluded	272
	Total	310
Gender	Male	16
	Female	22
Age	Median	60 (32-83)
Jaundice		30 (0-180)
	Total bilirrubin (mg/dl)	15.6 (0.2-38.0)
Endoscopic drainage		2
Surgical approach	CPD	17
	DPPP	21
	Length of time	480 (345-720)
	Blood transfusion (1-5 units)	28

CPD=classic pancreatoduodenectomy; PPPD=pylorus preserving pancreatoduodenectomy

Six patients were diagnosed with pancreatic fistula according to the definitions of the ISGPS^8^. Three patients had grade A fistulas, two grade B, and one grade C. According to this same Study Group, DGE was present in 33 patients^31^ (Table 2).

**TABLE 2:** ISGPS data - Pancreatic fistula and delayed gastric emptying

ISGPS		
Pancreatic fistula	A	3
	B	2
	C	1
Delayed gastric emptying	A	25
	B	6
	C	2

ISGPS=International Study Group in Pancreatic Surgery

Six patients had well differentiated tumors, 27 moderate differentiation, and five undifferentiated tumors. Median lymph node resection was eight (1-23). 23 patients had pN0 status and fifteen had pN+ status. Nine patients did not present ALI in the pathologists' reports (N0ALI-). Fourteen patients presented ALI and N0 status (N0ALI+). The remaining 15 patients presented pN+ status (N+ALI+). Twenty-three underwent gemcitabine-based adjuvant chemotherapy or chemoradiation. Seven were in the N0ALI+ group, five in the N0ALI- group and 11 were in the N+ALI+ group (Table 3).

**TABELA 3:** Pathology staging

Tumor	
Size (cm)	3.0 (1-15)
Differentiation:	
Well differentiated	6
Moderate	27
Undifferentiated	5
pT	
T1	2
T2	9
T3	26
T4	1
Margins	
pR0	23
pR1	15
Lymph nodes	
Dissected	8 (1-23)
pN0	23
pN+	15
Angiolymphatic invasion	
N0ALI-	9
N0ALI+	14
N+ALI+	15
Perineural invasion	
Yes	31
No	7

**FIGURE 1:**
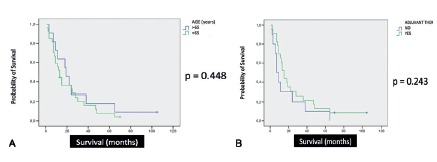
A) Age (65 years cut-off); B) adjuvant chemotherapy

**FIGURE 2:**
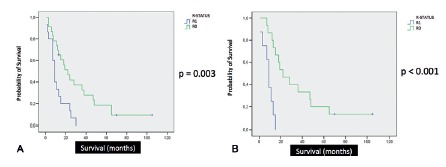
A) Status pR (univariate analysis); B) Status pR (bivariate analysis)

**FIGURE 3:**
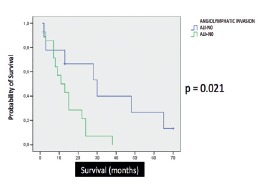
Angiolymphatic invasion in pN0 patients

Median overall survival was 13 months. There was no correlation between age at the time of operation and overall survival, even considering a 65-years-old cutoff in Log-Rank/Kaplan Meier curves (p=0.448, Figure 1A). The group of patients with adjuvant therapy did not show improved overall survival when compared to the group that did not receive chemotherapy or chemoradiation (p=0.243, Figure 1B). Patients with pR1 margin status had significantly poorer overall survival compared to those with pR0 margin status, both in univariate (p=0.003) and bivariate analysis (p<0.001) (Figures 2A and 2B). There was no correlation between tumor size and overall survival. This study did not show correlation between pT staging and the occurrence of angiolymphatic invasion (p=0.972). Considering patients with pN0 status and angiolymphatic invasion, this group had significantly poorer survival compared to the group without angiolymphatic invasion in univariate analysis (p=0.021/CI 95% - 10.489-19.511, Figure 3). There was no statistical difference in the number of lymph node resection between these groups (p=0.111). Perineural invasion was not significant (p=0.730). Patients without major postoperative complications did not have poorer overall survival (p>0.05). Jaundice time and weight loss (median=7 kg) prior to operation, hospital stay (median=12 days), and type of surgery (CPD vs. PPPD) did not correlate with overall survival.

Only three patients were alive and considered cured by the end of this research. Two of them were N0ALI- and one patient was pN+. Thirty patients died of documented systemic metastasis. Of seven patients with no ALI that died, two died of pneumonia, one of prostate cancer, and four due to systemic metastasis (hepatic or peritoneal carcinomatosis). All 14 N0ALI+ patients died. Only one died of sepsis in a febrile neutropenic patient in the course of chemotherapy. The remaining 13 developed peritoneal carcinomatosis, hepatic or pulmonary metastasis. In the N+ group (n=15), one died of complications of femur fracture and the remaining developed systemic metastasis (hepatic or peritoneal carcinomatosis).

## DISCUSSION

Around 90% of pancreatic tumors are ductal adenocarcinomas, and authors believe that once the genetic material of a pluripotent stem cell in adult pancreas is damaged and genetic changes accumulate, pancreatic intraepithelial neoplasia (PanIN) develop and may occasionally evolve into invasive pancreatic cancer^29^.

The most important risk factors include gender (slightly more common in men), age, cigarrete smoking and body mass index^13^. Some genetic syndromes are also related to a higher incidence of pancreatic cancer, such as familial adenomatous polyposis syndrome, Peutz-Jeghers syndrome, breast cancer familial syndrome, and hereditary nonpolyposis colorectal cancer syndrome^17^. None of these syndromes were detected in our group of patients.

The majority of pancreatic cancers (70-80%) are located in the head portion of the organ. Tumors from the body or tail of the pancreas are almost always unresectable because they usually grow silently^29^, with lack of symptoms.

Differently from the literature data, our patients were female in their majority^9^ ^,^ ^13^. This may be justified because, in our culture, men are usually more resistant to seek medical assistance. Moreover, the median time of jaundice prior to surgery was 43% higher than in the literature (30 days vs. 21 days)^18^ ^,^ ^26^.

In the first half of the 2000's decade, the majority of the operations performed were CPD. After this period, nearly all procedures consisted of PPPD. The reason was the publication of relevant papers in the early 2000's showing a tendency to better early postoperative outcomes favoring PPPD and similar oncological results compared to CPD^22^ ^,^ ^25^.

The occurrence of POPF was similar to the literature^4^ according to the definitions of the ISGPF^1^, with a prevalence of 15.8%. Moreover, considering only clinical relevant pancreatic fistula (ISGPS B or C), the prevalence was as low as 7.9%.

The great majority of our patients developed some degree of DGE according to the ISGPS definitions, (n=33, 86.8%), much more than the current literature, which ranges around 45%^30^. Although, 25 of these patients had grade A DGE. Such high prevalence could be explained by the current postoperative feeding protocol established in our group. All patients left the operating room with both a nasogastric tube for gastric decompression and enteral tube for feeding. Enteral feeding started between the 2^nd^ and 3^rd^ postoperative day, removing the gastric tube in the 5^th^, and began oral intake by the 6^th^. Therefore, our patients usually did not receive unlimited oral intake before the 7^th^ day.

Median tumor size was 3.0 cm, which is 15.4% larger than one of the largest casuistic ever published (2.6 cm)^3^. Most of all patients had pR0 resections, however the number of pR1 resections were not small (39.5%). Main limitation was the retroperitoneal margin or when the tumor was in the pancreatic surface and, for the patient that had the T4 tumor complete resection was not possible due to exuberant involvement of the common hepatic artery. Literature has demonstrated that sometimes pR1 resections account for at least 44% of the procedures, mainly when analyzing the retroperitoneal margins. This data actually shows a good quality of pathologic reporting. Nevertheless, palliative duodenopancreatectomy has shown to be acceptable with good postoperative quality of life and improved overall survival^6^ ^,^ ^19^.

Mostly, our patients had T3 tumors, especially because of intrapancreatic bile duct, duodenum, or peripancreatic soft tissue invasion, according to the UICC staging system. As it would be expected, overall survival decreased with the increment of the pT staging (exception for the single T4 resected case that survived 15 months).

In accordance to this research, even though authors have reported increased morbidity in elderly patients, mortality rates and overall survival range acceptable levels, with no significant differences when compared to younger patients^23^.

Although it is expected that tumor size (and pT staging) would influence the occurrence of angiolymphatic invasion and lymph node metastasis, it did not happen in this study, when compared to studies involving other abdominal tumors, such as gastric adenocarcinoma^33^. Also, our group did not perform extended lymphadenectomies^24^.

Diverging from other papers, in which perineural invasion was strictly correlated with poorer overall long-term survival^5^ ^,^ ^27^, in this study, this feature was not associated with better or worse prognosis.

It is believed that patients with ALI (lymphovascular, venous, or both) in resected N0 cephalic pancreatic adenocarcinoma behave not as they have a localized tumor, but as with systemic disease, with poor long-term overall survival. And lymphatic invasion within the tumor precedes regional lymph node metastasis. Most references found in literature regarding this feature as prognostic factors studied colorectal and breast cancers. Some others did study periampullary and pancreatic neoplasms. And all of them supported our hypothesis. Nevertheless, none of them individualized only patients with cephalic pancreatic adenocarcinoma and, more, only patients with pN0 status^4^ ^,^ ^5^ ^,^ ^7^ ^,^ ^8^ ^,^ ^12^ ^,^ ^14^ ^,^ ^15^ ^,^ ^16^ ^,^ ^20^. The intention of this study was to simplify the method and make it more accessible by not using biomarkers.

In the public health system, adequate treatment of the pancreatic adenocarcinoma becomes a challenge, not only for health care providers but also for the surgeons and patients^7^. Our institution is one of the few public tertiary hospitals specialized in pancreatic diseases in the city of São Paulo, Brazil. And the requirements become even larger once patients from other cities also seek for our assistance. Due to this large demand and limited resources, when most patients had reached our unit, they already presented systemic metastasis, unresectable disease, or did not meet clinical conditions to be submitted to resection with curative intent. This justifies that we have assisted a large number of patients with pancreatic adenocarcinoma, but only 12.3% (38/310) of them could be resected and followed adequately. The remaining were palliated either via endoscopic or surgical procedures, or died too prematurely.

Main limitation in this study was the total number of patients enrolled. Our casuistic did not allow more detailed statistical analysis due to the low number of patients in each group.

## CONCLUSION

This study evidenced that angiolymphatic invasion in pN0 resected cephalic pancreatic adenocarcinoma was determinant in overall survival. As an easy and accessible method, it should be encouraged in further prospective trials.
